# The complete mitochondrial genome of the bag-shelter moth *Ochrogaster lunifer *(Lepidoptera, Notodontidae)

**DOI:** 10.1186/1471-2164-9-331

**Published:** 2008-07-15

**Authors:** Paola Salvato, Mauro Simonato, Andrea Battisti, Enrico Negrisolo

**Affiliations:** 1 Department of Environmental Agronomy and Vegetal Productions-Entomology, University of Padova, Agripolis, Viale dell'Università 16, 35020 Legnaro, Italy; 2 Department of Public Health, Comparative Pathology and Veterinary Hygiene, University of Padova, Agripolis, Viale dell'Università 16, 35020 Legnaro, Italy

## Abstract

**Background:**

Knowledge of animal mitochondrial genomes is very important to understand their molecular evolution as well as for phylogenetic and population genetic studies. The Lepidoptera encompasses more than 160,000 described species and is one of the largest insect orders. To date only nine lepidopteran mitochondrial DNAs have been fully and two others partly sequenced. Furthermore the taxon sampling is very scant. Thus advance of lepidopteran mitogenomics deeply requires new genomes derived from a broad taxon sampling. In present work we describe the mitochondrial genome of the moth *Ochrogaster lunifer*.

**Results:**

The mitochondrial genome of *O. lunifer *is a circular molecule 15593 bp long. It includes the entire set of 37 genes usually present in animal mitochondrial genomes. It contains also 7 intergenic spacers. The gene order of the newly sequenced genome is that typical for Lepidoptera and differs from the insect ancestral type for the placement of *trnM*. The 77.84% A+T content of its α strand is the lowest among known lepidopteran genomes. The mitochondrial genome of *O. lunifer *exhibits one of the most marked C-skew among available insect Pterygota genomes. The protein-coding genes have typical mitochondrial start codons except for *cox1 *that present an unusual CGA. The *O. lunifer *genome exhibits the less biased synonymous codon usage among lepidopterans. Comparative genomics analysis study identified *atp6*, *cox1*, *cox2 *as *cox3*, *cob*, *nad1*, *nad2, nad4*, and *nad5 *as potential markers for population genetics/phylogenetics studies. A peculiar feature of *O. lunifer *mitochondrial genome it that the intergenic spacers are mostly made by repetitive sequences.

**Conclusion:**

The mitochondrial genome of *O. lunifer *is the first representative of superfamily Noctuoidea that account for about 40% of all described Lepidoptera. New genome shares many features with other known lepidopteran genomes. It differs however for its low A+T content and marked C-skew. Compared to other lepidopteran genomes it is less biased in synonymous codon usage. Comparative evolutionary analysis of lepidopteran mitochondrial genomes allowed the identification of previously neglected coding genes as potential phylogenetic markers. Presence of repetitive elements in intergenic spacers of *O. lunifer *genome supports the role of DNA slippage as possible mechanism to produce spacers during replication.

## Background

Animal mitochondrial genomes (mtDNAs) are usually circular molecules spanning 16–20 kbp that contain 13 protein-coding genes (PCGs), 2 ribosomal RNA and 22 transfer (tRNA) genes [1]. Non-coding control elements, that regulate the transcription and replication of the genome, are also present in mtDNAs [1,2]. Mitochondrial genomes are very important subject for different scientific disciplines including animal health, comparative and evolutionary genomics, molecular evolution, phylogenetics and population genetics. However, current knowledge on mtDNAs is very uneven as well exemplified by sequences available in GenBank that were obtained mostly from vertebrate taxa. Insects constitute the most species-rich class among animals with almost a million of taxa described to date [3]. Within the insects, the Lepidoptera (butterflies plus moths) order accounts for more than 160,000 species [4]. Despite this huge taxonomic diversity the existing information on lepidopteran mtDNA is very limited. Complete sequences have been determined for the two butterflies *Coreana raphaelis *and *Artogeia melete*, and for the seven moths *Adoxophyes honmai, Antheraea pernyi, Bombyx mori, Bombyx mandarina *and *Manduca sexta, Phthonandria atrilineata *and *Saturnia boisduvalii *[5-9] while near complete sequences exist for *Ostrinia furnacalis *and *Ostrinia nubilalis *[10] (Table 1). Current genomic knowledge of Lepidoptera is very scanty and the covered taxon-sampling is extremely poor and limited to six superfamiles among the 45–48 known, and to 9 families of the recognized 120 [4]. A better understanding of the lepidopteran mtDNA requires an expansion of taxon and genome samplings. We were able to fully sequence the mitochondrial genome of the bag-shelter moth *Ochrogaster lunifer*. The newly determined mtDNA is the first complete sequence for the Superfamily Noctuoidea, a very large assemblage that accounts for about 40% of all described Lepidoptera. [4]. In the present paper the *Ochrogaster *genome is described and compared with mtDNAs of other lepidopterans as well as pterygote Insecta.

**Table 1:** List of taxa analyzed in present paper

**Order**	**Family**	**Species**	**Acc. number**	**Reference**
**Orthoptera**	Acrididae	*Locusta migratoria*	NC_001712	[42]
	Acrididae	*Oxya chinensis*	NC_010219	Hang and Zhang, unpublished
	Tettigonidae	*Anabrus simplex*	NC_009967	[43]
	Tettigonidae	*Ruspolia dubia*	NC_009876	[44]
	Gryllotalpidae	*Gryllotalpa orientalis*	NC_006678	[45]
**Isoptera**	Rhinotermitidae	*Reticulitermes flavipes*	NC_009498	[13]
	Rhinotermitidae	*Reticulitermes hageni*	NC_009501	[13]
	Rhinotermitidae	*Reticulitermes virginicus*	NC_009500	[13]
	Rhinotermitidae	*Reticulitermes santonensis*	NC_009499	[13]
**Manthophasmatodea**	Mantophasmatidae	*Sclerophasma paresisense*	NC_007701	[46]
**Mantodea**	Mantidae	*Tamolanica tamolana*	NC_007702	[46]
**Blattaria**	Blattidae	*Periplaneta fuliginosa*	NC_006076	[47]
**Plecoptera**	Pteronarcyidae	*Pteronarcys princeps*	NC_006133	[48]
**Phthiraptera**	Boopidae	*Heterodoxus macropus*	NC_002651	[16]
	Philopteridae	*Bothriometopus macrocnemis*	NC_009983	[49]
	Philopteridae	*Campanulotes bidentatus*	NC_007884	[15]
**Hemiptera**	Aleyrodidae	*Aleurochiton aceris*	NC_006160	[14]
	Aleyrodidae	*Aleurodicus dugesii*	NC_005939	[14]
	Aleyrodidae	*Bemisia tabaci*	NC_006279	[14]
	Aleyrodidae	*Neomaskellia andropogonis*	NC_006159	[14]
	Aleyrodidae	*Tetraleurodes acaciae*	NC_006292	[14]
	Aleyrodidae	*Trialeurodes vaporariorum*	NC_006280	[14]
	Aphididae	*Schizaphis graminum*	NC_006158	[14]
	Cicadellidae	*Homalodisca coagulata*	NC_006899	Baumann and Baumann, unpublished
	Psyllidae	*Pachypsylla venusta*	NC_006157	[14]
	Aphrophoridae	*Philaenus spumarius*	NC_005944	[50]
	Reduviidae	*Triatoma dimidiata*	NC_002609	[51]
**Psocoptera**	Lepidopsocidae	Lepidopsocid sp. RS-2001	NC_004816	[52]
**Thysanoptera**	Thripidae	*Thrips imaginis*	NC_004371	[53]
**Coleoptera**	Cerambycidae	*Anoplophora glabripennis*	NC_008221	An et al., unpublished
	Chrysomelidae	*Crioceris duodecimpunctata*	NC_003372	[54]
	Elateridae	*Pyrophorus divergens*	NC_009964	[55]
	Lampyridae	*Pyrocoelia rufa*	NC_003970	[56]
	Tenebrionidae	*Tribolium castaneum*	NC_003081	[57]
**Lepidoptera**				
Tortricoidea	Tortricidae	*Adoxophyes honmai*	NC_008141	[7]
Bombycoidea	Saturniidae	*Antheraea pernyi*	NC_004622	Liu et al., unpublished
Bombycoidea	Bombycidae	*Bombyx mandarina*	NC_003395	[5]
Bombycoidea	Bombycidae	*Bombyx mori*	NC_002355	Lee et al., unpublished
Bombycoidea	Saturniidae	*Saturnia boisduvalii*	NC_010613	[9]
Geometroidea	Geometridae	*Phthonandria atrilineata*	NC_010522	Yang et al., unpublished
Papilionoidea	Pieridae	*Artogeia melete*	NC_010568	Hong et al., unpublished
Papilionoidea	Lycaenidae	*Coreana raphaelis*	NC_007976	[6]
Sphingoidea	Sphingidae	*Manduca sexta*	EU286785	[8]
**Noctuoidea**	**Notodontidae**	***Ochrogaster lunifer***	AM946601	**This paper**
Pyraloidea	Crambidae	*Ostrinia furnacalis*	NC_003368	[10]
Pyraloidea	Crambidae	*Ostrinia nubilalis*	NC_003367	[10]
**Diptera**	Ceratopogonidae	*Culicoides arakawae*	NC_009809	Matsumoto, unpublished
	Culicidae	*Aedes albopictus*	NC_006817	Ho et al., unpublished
	Culicidae	*Aedes aegypti*	NC_010241	Lobo et al., unpublished
	Culicidae	*Anopheles gambiae*	NC_002084	[58]
	Culicidae	*Anopheles quadrimaculatus *A	NC_000875	[59]
	Calliphoridae	*Cochliomyia hominivorax*	NC_002660	[60]
	Calliphoridae	*Lucilia sericata*	NC_009733	Cibrario et al., unpublished
	Calliphoridae	*Chrysomya putoria*	NC_002697	[61]
	Drosophilidae	*Drosophila melanogaster*	NC_001709	[62]
	Drosophilidae	*Drosophila mauritiana*	NC_005779	[63]
	Drosophilidae	*Drosophila sechellia*	NC_005780	[63]
	Drosophilidae	*Drosophila simulans*	NC_005781	[63]
	Drosophilidae	*Drosophila yakuba*	NC_001322	[64]
	Oestridae	*Dermatobia hominis*	NC_006378	Azeredo-Espin et al., unpublished
	Muscidae	*Haematobia irritans*	NC_007102	Lessinger et al., unpublished
	Nemestrinidae	*Trichophthalma punctata*	NC_008755	[65]
	Syrphidae	*Simosyrphus grandicornis*	NC_008754	[65]
	Tabanidae	*Cydistomyia duplonotata*	NC_008756	[65]
	Tephritidae	*Ceratitis capitata*	NC_000857	[66]
	Tephritidae	*Bactrocera carambolae*	NC_009772	Ye et al., unpublished
	Tephritidae	*Bactrocera dorsalis*	NC_008748	Yu et al., unpublished
	Tephritidae	*Bactrocera oleae*	NC_005333	[67]
	Tephritidae	*Bactrocera papayae*	NC_009770	Ye et al., unpublished
	Tephritidae	*Bactrocera philippinensis*	NC_009771	Ye et al., unpublished
**Hymenoptera**	Apidae	*Apis mellifera ligustica*	NC_001566	[68]
	Apidae	*Bombus ignitus*	DQ870926	[69]
	Apidae	*Melipona bicolor*	NC_004529	Silvestre and Arias, unpublished
	Vanhornidae	*Vanhornia eucnemidarum*	NC_008323	[70]

## Results and discussion

### Genome organization, structure and composition

**Figure 1:**
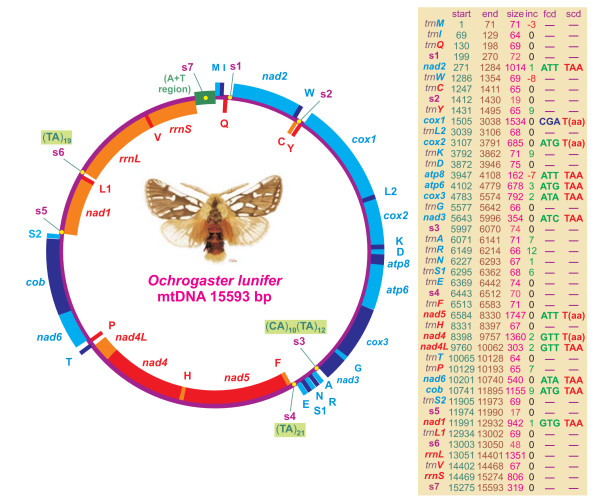
**Map of the mitochondrial genome of *O. lunifer***. Genes coded in the α strand (clockwise orientation) are blue or cyan colored. Genes coded in the β strand (anti-clockwise orientation) are red or orange colored. Alternation of colors was applied for clarity. Start, first position along α strand; end, last position along α strand; size, size of the sequence; inc, intergenic nucleotides; fcd, first codon; scd, stop codon. Incomplete stop codons are presented with parentheses. Negative inc values refer to overlapping nucleotides for genes located in the same or different strands. Gene names are the standard abbreviations used in this paper; tRNA genes are indicated by the single letter IUPAC-IUB abbreviation for their corresponding amino acid in the draw. s1–s7, intergenic spacers.

The mtDNA genome of *O. lunifer *is a circular molecule 15593 bp long. It includes the entire set of 37 genes usually present in animal mtDNAs [1], i.e., 13 PCGs, 22 tRNA genes, and 2 ribosomal genes (Figure 1). The mtDNA genome of *O. lunifer *contains also 7 intergenic spacers (s1–s7), spanning at least 15 bp, described in a paragraph below. Genes on the same strand are overlapped (e.g. *trnM *vs. *trnI*; *atp8 *vs. *atp6*), contiguous, separated by few nucleotides or by intergenic spacers (e.g. *nad3 *vs. *trnA*; *trnC *vs. *trnY*). Genes on opposite strands exhibit a similar behavior (Figure 1).

The *O. lunifer *mtDNA has the typical lepidopteran gene order [8,9] that differs from the ancestral gene order of insects [1] for the placement of *trnM*. In the ancestral type (e.g. *Drosophila yakuba *mtDNA) the order in the α strand is: A+T region, *trnI*, *trnQ*, *trnM*, *nad2*. In all lepidopteran mtDNAs, sequenced to date, the order is: A+T region, *trnM*, *trnI*, *trnQ*, *nad2 *which implies the translocation of *trnM *[5-11]. This placement of *trnM *is a molecular feature exclusive to lepidopteran mtDNAs. Further genome sequencing is necessary to establish if this feature is a mitochondrial signature of the whole order Lepidoptera.

The composition of the α strand of *O. lunifer *mtDNA is A = 6252 (40.09%), T = 5886 (37.75%), G = 1179 (7.56%) and C = 2276 (14.60%).

**Figure 2:**
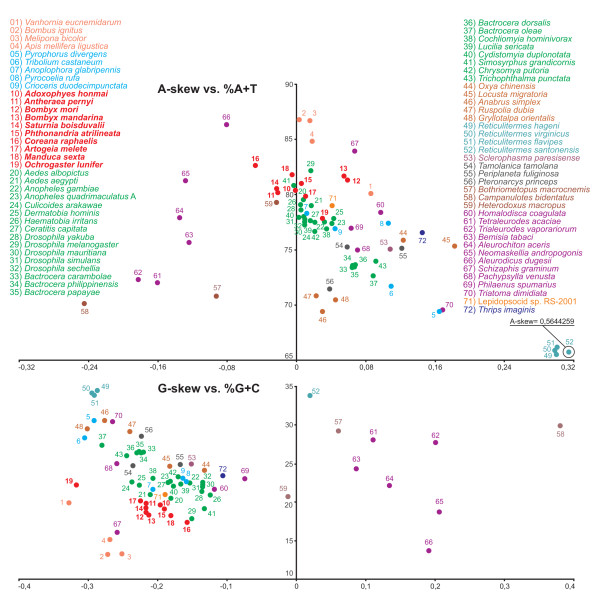
**A-skew vs. A+T% and G-skew vs. G+C% in the Pteryogota mtDNAs**. Values were calculated on α strands for full lengh mtDNA genomes. The X axis provides the skews values, while the Y axis provides the A+T/G+C values. Named of species are colored according to their taxonomic placement at Order level (see Table 1).

The A+T% and G+C% values for the α strand as well as the A- and G-skews [12] were calculated for all available complete mtDNA genomes of Pterygota and are presented in the scatter plots of Figure 2.

The average A+T% value for the analyzed mtDNAs set is 76. 63 ± 4.84. The highest A+T% values are shared by the mtDNAs of three bees (*Apis mellifera*, *Bombus ignitus *and *Melipona bicolor*) and two bugs (*Aleurodicus dugesii *and *Schizaphis graminum*). All lepidopteran mtDNAs but *O. lunifer *exhibit high A+T% values. The A+T content of *O. lunifer *mtDNA is 77.84% that represents the lowest value for lepidopteran complete mtDNAs [5-8,10]. The lowest A+T contents are found in the termite mtDNAs (*Reticulitermes *spp.). Extreme A+T values are also shared by species having highly re-arranged gene order [13]. However the possession of a re-arranged genome is not sufficient *per se *to have an A+T content drastically departing from the average (e.g. *Aleurochiton aceris *and *Bemisia tabaci*). The A+T values appear to be linked to taxonomic relatedness at low rank (i.e. genus, family) (e.g. species of *Drosophila*, species of *Bactrocera*, members of family *Apidae*). The relation is not true at higher ranks (i.e. superfamily; order) where patterns become inconsistent and the A+T content can be very different among species as exemplified by Hemiptera (*A. dugesii *vs.*Triatoma dimidiata)*.

The average A-skew is 0.04214 ± 0.11350 and most of pterygote mtDNAs are slightly to moderately A-skewed with values ranging from 0.00287 (*B. ignitus*) to 0.18247 (*Locusta migratoria*). The lepidopteran A-skews vary from -0.04748 (*C. raphaelis*) to 0.05872 (*B. mori*) with the *O. lunifer *mtDNA exhibiting a slight A-skew (0.03015). The *Reticulitermes *mtDNA genomes, having the lowest A+T% content, exhibit a very pronounced A-skew. Most marked T-skews are observed in the mtDNA genomes of *Campanulotes bidentatus *and *Trialeuroides vaporarium *that have low A+T% content and gene-orders different than insect ancestral gene order [1,14,15]. Gene order re-arrangement is not necessarily linked to strong A/T-skew as proved by the highly rearranged, but low skew, genome of *Heterodoxus macropus *[16].

The average G+C% content is 23.37 ± 4.84. The G+C% pattern among various species is obviously opposite to the A+T% thus it does not require further comments. More composite is the G/C-skew distribution. The average G-skew is -0.16006 ± 0.138235. Most of pterygota mtDNAs are C-skewed with G-skew values ranging from -0.32827 (*Vanhornia eucnemidarum*) to -0.01250 (*Heterodoxus macropus*). The main exception is represented by the mtDNA of bugs, while the highest G-skewed genome is that of *C. bidentatus*. Most of lepidopteran mtDNAs share very similar G-skew values that are included within the bulk of mtDNAs. The notable exception is represented by the newly determined mtDNA of *O. lunifer *that exhibits the second most pronounced C-skew (G-skew = -0.31751) among analyzed genomes.

G-skew can be markedly different even in species belonging to the same genus and having a very similar G+C content as well exemplified by *Reticulitermes santonensis *and *Reticulitermes virginicus *mtDNAs. The same reasoning applies at high taxonomic rank to the Hemiptera. The mtDNA of *C. bidentatus *exhibits very high A-skew and G-skew. However, this feature is not a general rule and extreme A-skew and G-skew are not necessarily reciprocally linked, as proved by species of genus *Reticulitermes *that exhibit very strong A-skews but not G-skews.

The list of currently available mtDNAs reveals that there is a strong bias in term of taxon sampling both at low and high taxonomic ranks within Pterygota. A direct consequence is that present knowledge of base composition and A/G skews reflects such biases and addition of a single taxon can change our view on these features. This point is well exemplified by the *O. lunifer *mtDNA that exhibits a A+T percentage different than other lepidopteran mtDNAs that share high A+T contents [8,9]. Thus a broad and more balanced taxon sampling appears to be a mandatory goal to investigate and identify general patterns for the parameters considered above.

### Protein-coding genes

The mtDNA of *O. lunifer *contains the full set of PCGs usually present in animal mtDNA. PCGs are arranged along the genome according to the standard order of Insects [1] (Figure 1). The putative start codons of PCGs are those previously known for animal mtDNA i.e. ATN, GTG, TTG, GTT [17] with the only exception represented by the CGA start codon of *cox1 *gene. This non-canonical putative start codon is found also in the butterfly *A. melete *and in the moths *A. honmai, B. mori, B. mandarina*, *M. sexta *and *P. atrilineata *[5-8]. In the butterfly *C. raphaelis *the tetranucleotide TTAG is the putative start codon [6] and the six nucleotide TATTAG has been suggested as putative start codon for the moths *O. nubilalis *and *O. furnicalis *[10]. An unusual start codon for *cox1 *gene is known in various arthropod mtDNA [e.g. [18]].

The *cox1*, *cox2*, *nad5*, and *nad4 *genes of *O. lunifer *mtDNA have incomplete stop codons. The presence of incomplete stop codons is a feature shared with all lepidopteran mtDNAs sequenced to date [5-10] and more in general with many arthropod mtDNAs [1].

The *atp8 *and a *atp6 *of *O. lunifer *are the only PCGs having a seven nucleotides overlap (Figure 1). This feature is common to all lepidopteran mtDNA genomes known [5-10] and is found in many animal mtDNAs [1].

**Figure 3:**
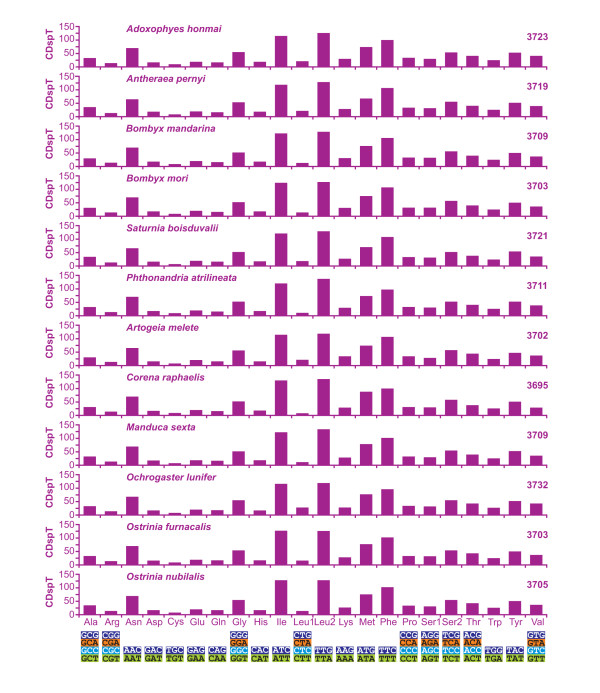
**Codon distribution in lepidopteran mtDNAs**. Numbers to the left refer to the total number of codon. CDspT, codons per thousands codons. Codon Families are provided on the x axis.

**Figure 4:**
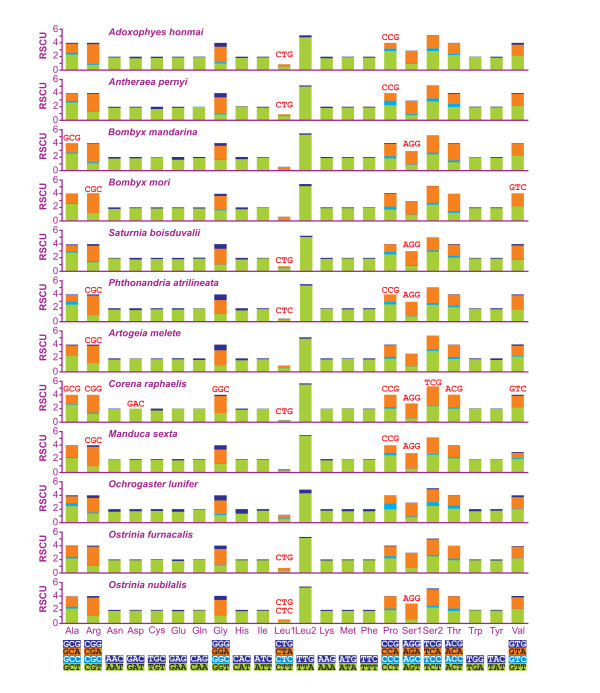
**Relative Synonymous Codon Usage (RSCU) in lepidopteran mtDNAs**. Codon Families are provided on the x axis. Red-colored codon, codon not present in the genome. Codon Families are provided on the x axis.

The abundance of codon families and Relative Synonymous Codon Usage (RSCU) [19] in PCGs were investigated for all available lepidopteran mtDNAs and the results are summarized in Figures 3 and 4. All first codons as well as stop codons, complete and incomplete, were excluded from the analysis to avoid biases due to unusual putative start codons and incomplete stop codons.

Total number of non-stop codons (CDs) used by the 12 analyzed mtDNAs is very similar ranging from 3695 of *C. raphaelis *to 3732 of *O. lunifer*. The codon families exhibit a very similar behavior among considered species. The eight codon families with at least 50 CDs per thousand CDs (Leu2, Ile, Phe, Met, Asn, Ser2, Gly, Tyr) encompass an average 65.82% ± 1.20% of all CDs. The three families with at least 100 CDs per thousand CDs (Leu2, Ile, Phe) account for an average 35.36% ± 0.98% of all CDs (Figure 3). The A+T rich CDs are favored over synonymous CDs with lower A+T content as proved by RSCU results (Figure 4). This point is well exemplified by the Leu2 family where the TTA codon accounts for the large majority of CDs in the family (see below). Invertebrate mitochondrial code includes 62 amino-acid encoding codons [1]. Among the 12 analyzed genomes the total number of used codons results to be directly linked to the A+T content. The *C. raphaelis *mtDNA, having the highest A+T% content (see Figure 2) uses 52 codons, and never utilized the 10 G+C rich codons listed in Figure 2. Conversely, *O. lunifer *mtDNA, characterized by the lowest A+T% among considered lepidopteran genomes, uses all 62 codons. Differences in the number of used CDs are present between species of the same genus (e.g. *B. mandarina *vs. *B. mori*) even if the discrepancies appear circumscribed to G+C rich CDs with very limited use (e.g. GCG and CGC). The Leu1 (average = 11.73 ± 3. 82%) and Leu2 (average = 88.44 ± 3.89%) codon families are very differently represented in lepidopteran PCGs while Ser1 (average = 34.95 ± 3.67%) and Ser2 (average = 64.05 ± 1.09%) exhibit a more balanced composition.

Four amino acid residues (Leu, Ile, Phe and Ser) account for more than 44.50% (average = 45.68 ± 0.58%) of all residues forming the 13 mitochondrial proteins. The Leu and Ile amino acids share hydrophobic lateral chains, Phe is also hydrophobic and Ser exhibits an aliphatic behavior [20] thus their massive presence is striking but not surprising for membrane proteins.

**Figure 5:**
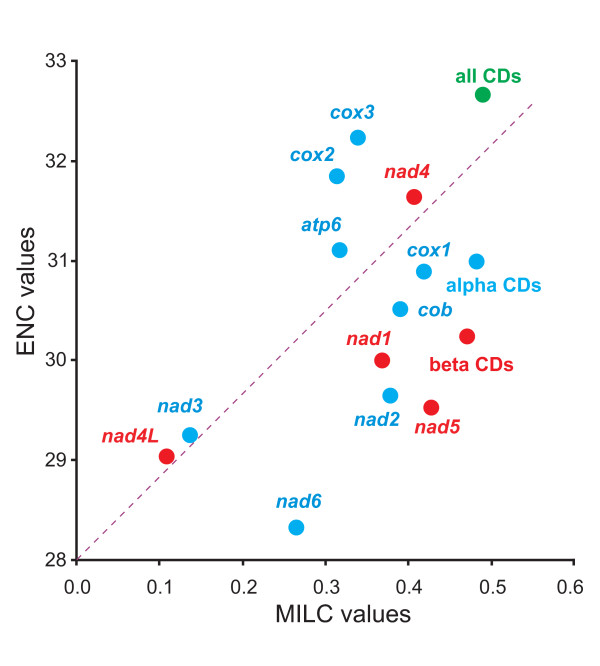
**Scatter plot graphic of MILC vs. ENC calculated for PCGs of lepidopteran mtDNAs**. Dots correspond to average values calculated for different genes. PCGs on α strand are blue-colored, PCGs on β strand are red colored. All pooled PCGs are presented as a green dot plot. Genes nomenclature as in main text.

Codon usage by single PCGs was investigated by calculating the two indices ENC (Effective number of codon used) [21] and MILC (Measure Independent of Length and Composition) [22]. Both indices, based on different approaches [21,22] provide a measure of codon variability of PCGs. The ENC and MILC estimate the codon variability in a way that allows comparison among sequences having different lengths as is the case of various PCGs. Genes exhibiting a higher diversity in codon usage have generally a higher number of variable sites, a prerequisite to be potential phylogenetic markers. Thus the use of ENC and MILC scores, according to the new approach presented in this paper, is a way to study PCG sequences variability on a codon perspective. The best scores of both indices should allow to identify the more diverse PCGs in a approach complementary to the usual method based on evolutionary distances among orthologous sequences (e.g. [8]). The assessment of genetic variability is an interesting point. Indeed some PCGs are standard marker for species recognition [23] or have been extensively used as phylogenetic markers in Lepidoptera while others have received so far limited or no attention. Understanding the genetic diversity of each PCG is a prerequisite to determine its phylogenetic usefulness. The ENC and MILC values were calculated for all PCGs but *atp8 *that contains too a few codons to get reliable ENC/MILC estimations [22]. Calculations were extended also to all 13 PCGs pooled as well as to the pooled PCGs belonging to α and β strands respectively. The scatter plot analysis is provided in Figure 5. As expected the greatest diversity in codon usage is found when all codons are considered. Good codon diversity is found also when all PCGs of α or β strands are considered. More interesting is the behavior of single genes. In this latter case sequences well established as phylogenetic markers (i.e. *cox1*, *cob*, *nad5*, and *cox2*) are intermixed with PCGs poorly or not considered by researchers (e.g. *cox3*, *nad4*, *nad1*, *nad2*). Our results suggest that the neglected PCGs should be considered as potential markers thus extending the number of mtDNA PCGs sampled for population as well as phylogenetic markers. Findings, based on codon diversity, must be integrated with direct comparisons of sequences [8] that allow to better define the optimal task that each gene can perform i.e. to be used at low taxonomic level or at high taxonomic level.

### Transfer and ribosomal RNA genes

**Figure 6:**
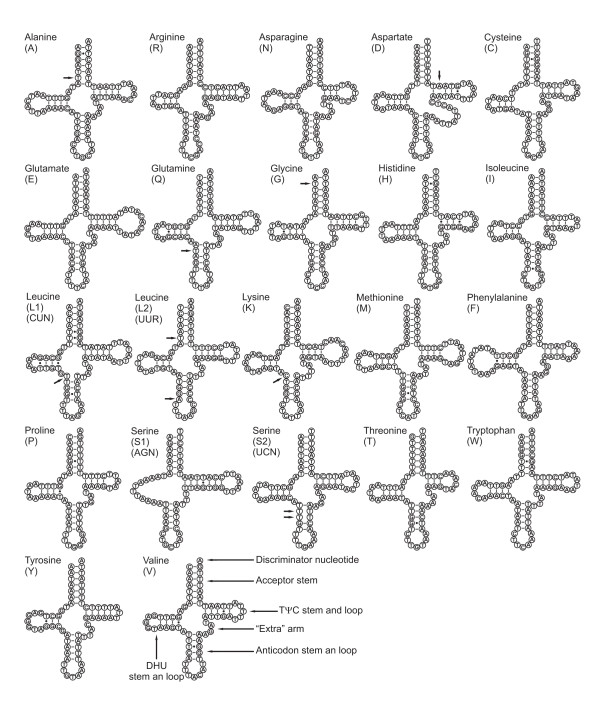
Secondary structures of transfer tRNAs in *O. lunifer *mtDNA.

*Ochrogaster *genome has the characteristic 22 tRNAs set (Figure 6) present in most of animal mtDNAs [1]. All tRNAs present the typical clover leaf secondary structure but *trnS1 *lacks the DHU stem. This feature is shared with the *C. raphaelis *mtDNA [6] but is not a general feature of lepidopteran mtDNA as proved by *A. honmai *that has all tRNAs with a complete clover leaf structure [7]. In general, the lack of DHU arm in *trnS1 *is a common condition in metazoan mtDNAs [24].

The *trnA*, *trnD*, *trnG*, *trnK*, *trnL1*, *trnL2*, *trnQ*, and *trnS2 *of *Ochrogaster *mtDNA show mismatches in their stems. Mismatches are located mostly in the acceptor and anticodon stems with a single exception represented by *trnD *that exhibits the mismatch on the TΨC stem. Mismatches on tRNA stems are known also for the *trnA*, *trnL1*, *trnL2*, and *trnQ*, of *C. raphaelis *[6]. Mismatches observed in tRNAs are corrected through RNA-editing mechanisms that are well known for arthropod mtDNA [e.g. [24]].

Preliminary analysis performed on *rrnL *and *rrnS *of *O. lunifer *revealed that these genes are capable of folding into structures (data not shown) similar to those already produced for lepidopteran mitochondrial ribosomal subunits [8,25,26]. Further studies, that extend the taxon sampling, are currently in progress in our lab to better define *rrnL *and *rrnS *structures within the Thaumetopoeinae subfamily that includes also *O. lunifer*.

### Non coding regions

**Figure 7:**
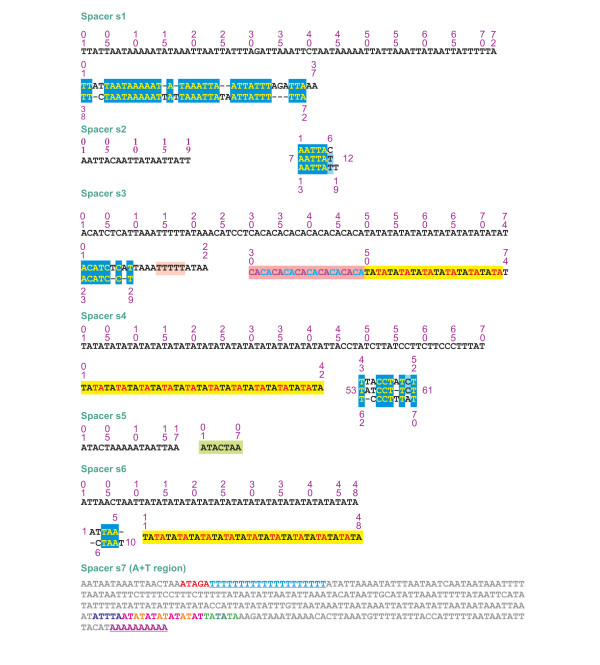
**Genomic spacers in the mtDNA of O. lunifer**. The sequences of spacers are those present in the α strand.

The mtDNA genome of *O. lunifer *contains 7 intergenic spacers (s1–s7) spanning at least 15 bp (Figures 1 and 7). The features of s1–s7 spacers are presented below with reference to the α strand for orientation and sequence motifs description.

The s1 spacer, located between *trnQ *and *nad2*, appears to be the result of a duplicated segment (Figure 7). The s1 spacer is present in all 12 lepidopteran mtDNAs so far sequenced while it is absent in other insects [8]. While the genomic location is constant the sequence divergence is high among species [8]. Further investigation with a broad taxon sampling within the Lepidoptera is necessary to assess if the s1 spacer is a constant molecular signature of lepidopteran mtDNA.

The s2 spacer, placed between *trnC *and *trnY*, derives from the triplication of a six nucleotides motif with minor changes (Figure 7). An 11 bp spacer between *trnC *and *trnY *is found also in the mtDNA of *A. melete *and shares the ACAATT motif with the s2 spacer of *O. lunifer*. Because no other known lepidopteran mtDNA exhibits such a spacer its presence in *A. melete *and *O. lunifer *has to be interpreted as the result of independent events.

Spacer s3, located between *nad3 *and *trnA*, exhibits a partial duplicated segment and a poly-T motif within the first 30 nt. The second half of s3 spacer is characterized by two microsatellite repeats (CA)_10_(TA)_12_. Spacers having the same genomic location, and containing TA microsatellites are found also in *B. mori *and *B. mandarina *mtDNA genomes.

Spacer s4, inserted between *trnE *and *trnF*, contains a 5' microsatellite (TA)_23_, while the 3' half seems to be the triplication of a 10 nucleotides motif with some changes (Figure 7). A spacer characterized by a different motif (TATTA)_31_, but having the same genomic placement, is found in the *A. honmai *mtDNA genome.

The spacer s5, located between *trnS2 *and *nad1*, contains the ATACTAA motif which is conserved across the Lepidoptera order [8]. This motif is possibly fundamental to site recognition by the transcription termination peptide (mtTERM protein) [2]. Spacer s5 is present in most insect mtDNAs even if the nucleotide sequence can be quite divergent [8].

The s6 spacer is located between *trnS2 *and -*rrnL *and exhibits a di-nucleotide microsatellite (TA)_19 _directly in contact with the 3' end of *rrnL *gene. To date spacer s6 is known only for the mtDNA of *O. lunifer*.

The s7 spacer coincides with the A+T region. Several features common to the Lepidoptera A+T region [8] are present in the s7 spacer. The O_R_β (origin of the β strand replication) is located 21 bp downstream from *rrnS *gene in *B. mori *[27]. It contains the motif ATAGA followed by an 18 bp poly-T stretch. A very similar pattern occurs in *O. lunifer *where the ATAGA motif is located 17 bp downstream from *rnnS *gene and is followed by a 20 bp poly-T stretch (Figure 7). A microsatellite-like (AT)_7_(TA)_3 _element preceded by the ATTTA motif is present in the 3' third of *O. lunifer *s7 spacer. The presence of a microsatellite preceded by the ATTA motif is also a feature found in the A+T regions of other Lepidoptera [8]. Finally a 10 bp poly-A is present immediately upstream *trnM*. This poly-T (in the β strand) element is still a common feature of the A+T region in Lepidoptera [8,28]. No large repeated segments were detected in the A+T region of *O. lunifer*. This arrangement is consistent with other lepidopteran A+T regions while markedly contrasts with patterns observed in other insect orders [8,29].

Intergenic spacers containing repeated elements are scattered all over the lepidopteran mtDNAs while repeated elements are restricted mostly to the A+T region in other insects [8]. Most parts of spacers of *O. lunifer *are made by repeated motifs. Predominance of repeated elements suggest that mtDNA expansion can be achieved through a miss-pairing duplication mechanism, i.e. DNA slippage, during genome replication. Several intergenic spacers are restricted to a single butterfly/moth species and have not counterparts even within Lepidoptera. Thus it is plausible to suggest that spacers production occurs independently and recursively within Lepidoptera. It remains unknown while this feature is so prominent in moths and butterflies and apparently limited, reduced or absent in other insect mtDNAs sequenced to date. This behavior requires further investigation provided that mtDNA intergenic spacers are found in non-insect Arthropoda as well as other animal phyla [e.g. [18,30]].

## Conclusion

The mitochondrial genome of *O. lunifer *is the first sequenced mtDNA for a representative of the Noctuoidea a superfamily that includes about 40% of all described lepidopteran species. The newly determined genome shares the gene order, the presence of intergenic spacers, and other features with previously known lepidopteran genomes. The placement of *trnM *immediately after the A+T region results to be an exclusive molecular signature of all lepidopteran mtDNAs sequenced to date. Further genome sequencing will establish if this feature characterizes the whole order Lepidoptera. The mtDNA of *O. lunifer *exhibits a peculiar low A+T content and marked C-skew. Compared to other lepidopteran genomes it is less biased in synonymous codon usage. Comparative analysis on codon usage among lepidopteran mitochondrial genomes identified *atp6*, *cox1*, *cox2, cox3*, *cob*, *nad1*, *nad2, nad4*, and *nad5 *as potential markers for phylogenetic and population genetic studies. Most of the genes listed above have been previously neglected for the tasks suggested here. The massive presence of repetitive elements in intergenic spacers of *O. lunifer *genome lead us to suggest an important role of DNA slippage as possible mechanism to produce spacers during replication.

## Methods

### Sample origin and DNA extraction

An ethanol-preserved larva specimen of *Ochrogaster lunifer *collected in Australia (Suburb of Kenmore, Queensland, 25th February 2005) by Myron P. Zalucki (University of Queensland) was used as starting material for this study. Total DNA was extracted by applying a salting-out protocol [31]. Quality of DNA was assessed through electrophoresis in a 1% agarose gel and staining with ethidium bromide.

### PCR amplification and sequencing of *Ochogaster lunifer *mtDNA

PCR amplification was performed using a mix of insect universal primers [32,33] and primers specifically designed on the *O. lunifer *sequences. For a full list of successful primers as well as PCR conditions see Additional file 1. The PCR products were visualized in electrophoresis in a 1% agarose gel and staining with ethidium bromide. Each PCR product represented by a single electrophoretic band was purified with the ExoSAP-IT kit (Amersham Biosciences) and directly sequenced. Sequencing of both strands was performed at the BMR Genomics service (Padova, Italy) on automated DNA sequencers mostly employing the primers used for PCR amplification.

### Sequence assembly and annotation

The mtDNA final consensus sequence was assembled using the SeqMan II program from the Lasergene software package (DNAStar, Madison, WI). Genes and strands nomenclature used in this paper follows Negrisolo et al. [18].

Sequence analysis was performed as follows. Initially the mtDNA sequence was translated into putative proteins using the Transeq program available at the EBI web site. The true identity of these polypeptides was established using the BLAST program [34,35] available at the NCBI web site. Gene boundaries were determined as follows. The 5' ends of PEGs were inferred to be at the first legitimate in-frame start codon (ATN, GTG, TTG, GTT; [17]) in the open reading frame (ORF) that was not located within the upstream gene encoded on the same strand. The only exception was *atp6*, which has been previously demonstrated to overlap with its upstream gene *atp8 *in many mtDNAs [17]. The PCG terminus was inferred to be at the first in-frame stop codon encountered. When the stop codon was located within the sequence of a downstream gene encoded on the same strand, a truncated stop codon (T or TA) adjacent to the beginning of the downstream gene was designated as the termination codon. This codon was thought to be completed by polyadenylation to a complete TAA stop codon after transcript processing. Finally pair-wise comparisons with orthologous proteins were performed with ClustalW program [36] to better define the limits of PCGs.

Irrespectively of the real initiation codon, a formyl-Met was assumed to be the starting amino acid for all the proteins as previously proved for other mitochondrial genomes [37,38].

The transfer RNA genes were identified using the tRNAscan-SE program [39] or recognized manually as sequences having the appropriate anticodon and capable of folding into the typical cloverleaf secondary structure [17].

The boundaries of the ribosomal *rrnL *gene were assumed to be delimited by the ends of the *trnV*-s6 pair. The 3' end of *rrnS *gene was assumed to be delimited by the start of *trnV *while the 5'end was determined through comparison with orthologous genes of other Lepidoptera so far sequenced.

### Genomic analysis

Nucleotide composition was calculated with the EditSeq program included in the Lasergene software package. The GC-skew = (G-C)/(G+C) and AT-skew = (A-T)/(A+T) were used [12] to measure the base compositional difference between the different strands or between genes coded on the alternative strands. The Relative Synonymous Codon Usage (RSCU) values were calculated with MEGA 4 program [40].

The codon usage by analyzed genomes was investigated by calculating the two indices ENC (Effective Number of Codon used) [21] and MILC (Measure Independent of Length and Composition [22]. ENC and MILC values were calculated with the INCA 2.1 program [41].

## Abbreviations

mtDNA: mitochondrial DNA; *atp6 *and *atp8*: ATP synthase subunits 6 and 8; *cob*: apocytochrome b; *cox1*-*3*: cytochrome c oxidase subunits 1–3; *nad1*-*6 *and *nad4L*: NADH dehydrogenase subunits 1–6 and 4L; *rrnS *and *rrnL*: small and large subunit ribosomal RNA (rRNA) genes; *trnX*: transfer RNA (tRNA) genes, where X is the one-letter abbreviation of the corresponding amino acid; s1–s7: mitochondrial genomic spacers; A+T region: the putative control region; PCG: protein coding gene; RSCU: Relative Synonymous Codon Usage; ENC, MILC: Measure Independent of Length and Composition; aa: amino acids; nt: nucleotides; bp: base pairs.

## Authors' contributions

PS and MS carried out the molecular experiments. AB and EN designed and coordinated all experiments. EN performed the genomic analyses. All authors contributed to the manuscript and then read and approved the final version.

## Supplementary Material

Additional file 1 Additional file 1. List of primers and PCR conditions used in the sequencing of *Ochogaster lunifer *mtDNA. Click here for file
