# Supplementary material for: The complete mitochondrial genome of the bag-shelter moth Ochrogaster lunifer (Lepidoptera, Notodontidae)
Source: BMC Genomics. 2008 Jul 15;9:331. doi: 10.1186/1471-2164-9-331 (PMC2488359; doi:10.1186/1471-2164-9-331)
Supplement: Additional file 1 — List of primers and PCR conditions used in the sequencing of Ochogaster lunifer mtDNA. [file 1471-2164-9-331-S1.pdf]

Additional file 1 – List of primers and PCR conditions used in the sequencing of *Ochogaster lunifer* mtDNA (Salvato et al)

	Primer Forward		Primer Reverse		AT		
**name**	**5'- sequence-3'**	**name**	**5'- sequence-3'**	**MIX**	**°C**	**EXT**	**CYCL**
**TFJ6400**	TAA TAT CTT CAA TRT YAA RCT CT	**071105_Rev3**	TTT TTA GCC GGA TTT TAT TCT	**1**	**54**	**2'**	**35**
**TFJ6400**	TAA TAT CTT CAA TRT YAA RCT CT	**OC_nad5r**	TTA TGG CTG GTA TTT CTG CT	**1**	**54**	**2'**	**40**
**TNJ6172**	AGA GGT ATA TCA CTG TTA ATG A	**HCnad5r2**	GCT GGG ATA TTA ACG GCT TT	**1**	**61**	**2'**	**40**
**N5J7806**	GAA ACT AAA CCC AAA CCA TCT CA	**N4N8727**	AAA TCT TTR ATT GCT TAT TCW TC	**2**	**62**	**2'**	**35**
**HCnad4f1**	ATC TAT ACA CTC CGC AAT AA	**HCnad6r1**	AAA GTT CGT TAG AAG CAA TAC	**2**	**60**	**2'**	**35**
**n4n6for**	CCA ATT AAC ATG GGT AAA GA	**n4n6rev**	TTG GGA TAT GAT GGT TTT TG	**2**	**58**	**1'30''**	**35**
**Oc3dfor**	GTC TTG TAA AYC AAA AWT AAG A	**Oc3adrv**	TAG CAA TAA TAA AGG GGA AA	**2**	**50**	**2'**	**35**
**N4J9172**	CGC TCT GGY TGR TAA CCY CA	**CBN11010**	TAT CTA CAG CAA ATC CCC CTC A	**2**	**62**	**2'**	**35**
**CBJ10933**	TAT GTA CTA CCA TGA GGA CAA ATA TC	**CBN11367**	ATT ACA CCT CCT AAT TTA TTA GGA AT	**2**	**52**	**1'**	**30**
**CBJ10933**	TAT GTA CTA CCA TGA GGA CAA ATA TC	**TS1N11683**	TAT TTC TTT ATT ATG TTT TCA AAA C	**1**	**58**	**3'**	**30**
**cobfor**	TTC CAC CCA TAT TTT TCC TTT A	**N1N12595**	GTA GCA TTT TTA ACT TTA TTA GAA CG	**1**	**58**	**3'**	**30**
**OC8ff_For**	CTA AAG CCA ATC TTA CTT CG	**OC8rr_Rev**	AAG TCT AAT CTG CCC ACT G	**2**	**58**	**3'**	**35**
**16Sfor**	GGT CGC AAA CTC TCT CTT TTA T	**12Srev**	ATG ATT TGG CGG TAT TTT AGT T	**1**	**52**	**1'30''**	**35**
**OC10ff_For**	GCC GAA TTC CTC AAA ATT A	**OC10rr_Rev**	TTA AAT CAA ATC AAG ATG CAG A'	**2**	**58**	**3'**	**35**
**SRJ14197**	GTA CCY CTA CTT TGT TAC GAC TT	**TMN200**	TCC TTT ATA TTT GAG GTA TGA RCC	**2**	**62**	**2'**	**35**
**OC2ff_For**	TTT GTA TAA CCG CAA CTG CT	**OC2rr_Rev**	TGA AGC AAT AGA TTG GGT TAA	**2**	**58**	**3'**	**35**
**Simon 12Sfor**	TAG GGT ATC TAA TCC TAG TT	**SimonMetrev**	TGG GGT ATG AAC CCA AAA GC	**1**	**56**	**1'30''**	**35**
**T1J34**	GCC TGA ATA AAG GRT TAY YCT GAT A	**TYN1433**	GGC TGA ATA ATA AGC GAT AAA TTG TAA A	**2**	**54**	**2'**	**35**
**n2c1for**	GGA TTT TTC CCT AAA TGA ATT G	**n2c1rev**	AAA AGC ATG TGC AGT TAC AA	**2**	**62**	**2'**	**35**
**LCO1490**	GGT CAA CAA ATC ATA AAG ATA TTG G	**HCO2198**	TAA ACT TCA GGG TGA CCA AAA AAT CA	**1**	**48**	**1'**	**35**
**c1for**	CCT CTC ATT CGA CCA AAT ACC	**c1rev**	TCG GGG TAA TCT GAA TAA CGA	**2**	**62**	**2'**	**35**

AT, annealing temperature; EXT, extension time; CYCL, number of PCR cycles; azure primer, primer located in the α strand of mitochondrial genome; red primer, primer located in the β strand of mitochondrial genome.

Primer Forward		Primer Reverse			TM		
**name**	**5'- sequence-3'**	**name**	**5'- sequence-3'**	**MIX**	**°C**	**EXT**	**CYCL**
**c1L2for**	TCA GAT TAC CCC GAC TCA TA	**c1L2rev**	GGT TAG ATC AAG TAG CCA TTT C	**2**	**58**	**2'**	**35**
**c2a6for**	GGA GTA AAA GTA GAC GCA AAC C	**c2a6rev**	AAA AGT GGA AAC GAT TAG GG	**2**	**62**	**2'**	**35**
**TKJ3790**	CAT TAG ATG ACT GAA AGC AAG TA	**C3N4908**	CGT GAA AYA TCT CGT CAT CAT TG	**2**	**62**	**2'**	**35**
**HCatp6f1**	ACT CTC AAG GAT CAA CAA TC	**HCcox3r1**	ATA ACG TGT AAA CCG TGG A	**2**	**58**	**1'30''**	**35**

AT, annealing temperature; EXT, extension time; CYCL, number of PCR cycles; azure primer, primer located in the α strand of mitochondrial genome; red primer, primer located in the β strand of mitochondrial genome.

	**Mix1**	**Mix2**
buffer 10X	4µl	4µl
$MgCl_2$ 25mM	2µl	1,5µl
dNTP's 2mM	1µl	1µl
primer fw 10µM	1µl	0,8µl
primer rw 10µM	1µl	0,8µl
Taq (5U/µl)	0,2µl	0,2µl
DNA sample	2µl	2µl
H_2O	8,8µl	9,7µl
**TOTAL**	**20µl**	**20µl**
